# Investigation of How Corneal Densitometry Artefacts Affect the Imaging of Normal and Keratoconic Corneas

**DOI:** 10.3390/bioengineering11020148

**Published:** 2024-02-01

**Authors:** Rami Alanazi, Louise Pellegrino Gomes Esporcatte, Lynn White, Marcella Q. Salomão, Bernardo T. Lopes, Renato Ambrósio Jr., Ahmed Abass

**Affiliations:** 1 Department of Materials, Design and Manufacturing Engineering, School of Engineering, University of Liverpool, Liverpool L69 3GH, UK; 2 Rio de Janeiro Corneal Tomography and Biomechanics Study Group, Rio de Janeiro 20520-050, Brazil; 3 Instituto de Olhos Renato Ambrósio, Rio de Janeiro 20520-050, Brazil; 4 Department of Ophthalmology, Federal University of São Paulo, São Paulo 04017-030, Brazil; 5 Research and Development Department, LWVision, Leicester LE18 1DF, UK; 6 Instituto Benjamin Constant, Rio de Janeiro 22290-255, Brazil; 7 Ophthalmology Eye Clinic, Alder Hey Children’s NHS Foundation Trust, Liverpool L12 2AP, UK; 8 Department of Ophthalmology, Federal University the State of Rio de Janeiro, Rio de Janeiro 22290-240, Brazil; 9 Brazilian Study Group of Artificial Intelligence and Corneal Analysis-BrAIN, Rio de Janeiro & Maceió, Rio de Janeiro 20520-050, Brazil

**Keywords:** eye, cornea, densitometry, artefacts, keratoconus, KC cone, KC area of pathology

## Abstract

Purpose: To investigate corneal densitometry artefacts found in Pentacam Scheimpflug scans and their potential effect on assessing keratoconic (KC) corneas compared to normal (N) corneas. Methods: The current study utilises Pentacam data of 458 N eyes, aged 35.6 ± 15.8 (range 10–87), referred to as the “N group”, and 314 KC eyes, aged 31.6 ± 10.8 (range 10–72), referred to as the “KC group”, where densitometry data were extracted and analysed via a custom-built MATLAB code. Radial summations of the densitometry were calculated at diameters ranging from 0.5 mm to 5.0 mm. The minimum normalised radial summation of densitometry (NRSD) value and angle were determined at each diameter and then linked. KC cone locations and areas of pathology were determined, and a comparison between N and KC groups was carried out both within the averaged area of pathology and over the corneal surface. Results: Joining minimum NRSD trajectory points marked a clear distortion line pointing to the nasal-superior direction at 65° from the nasal meridian. The findings were found to be independent of eye laterality or ocular condition. Consistency was detected in the right and left eyes among both the N and KC groups. The location of the KC cone centre and the area of pathology were determined, and the densitometry output was compared both within the area of pathology and over the whole cornea. When the average densitometry was compared between N and KC eyes within the KC area of pathology, the N group recorded a 16.37 ± 3.15 normalised grey-scale unit (NGSU), and the KC group recorded 17.74 ± 3.4 NGSU (*p* = 0.0001). However, when the whole cornea was considered, the N group recorded 16.71 ± 5.5 NGSU, and the KC group recorded 15.72 ± 3.98 NGSU (*p* = 0.0467). A weak correlation was found between the Bad D index and NGSU when the whole measured cornea was considered (R = −0.01); however, a better correlation was recorded within the KC area of pathology (R = 0.21). Conclusions: Nasal-superior artefacts are observed in the densitometry Pentacam maps, and analysis shows no significant differences in their appearance between N or KC corneas. When analysing KC corneas, it was found that the cone positions are mostly on the temporal-inferior side of the cornea, opposite to the densitometry artefact NRSD trajectory. The analysis suggests that the corneal densitometry artefacts do not interfere with the KC area of pathology as it reaches its extreme in the opposite direction; therefore, weighting the densitometry map to increase the contribution of the inferior-temporal cornea and decreasing that of the superior-nasal area would improve the classification or identification of KC if densitometry is to be used as a KC metric.

## 1. Introduction

Advancements in non-invasive imaging techniques are increasingly being used in diagnosing and monitoring ocular conditions, including ultrasonic elastography (UE), Optical coherence elastography (OCE), Optical Coherence Tomography (OCT), or Scheimpflug systems [1,2,3,4,5,6].

Corneal densitometry is an imaging technology used by instruments such as OCT or Scheimpflug systems to measure the amount and distribution of backscattered light from different zones of the cornea. While this technique can provide valuable information about the cornea’s health, it is vital to be aware of potential artefacts or errors that can affect the accuracy of the results. These artefacts can arise from relatively manageable issues such as eye movement, poor fixation, scatter from light in the measurement room, and out-of-date instrument calibration. More significantly, they can also be caused by subject-related factors such as tear film instability and eye tilt [7] or, in the case of OCT densitometry, epithelial speckle [8].

Corneal densitometry is increasingly being used as a clinical tool to monitor and assist in the diagnosis of ocular conditions such as high myopia [9,10], corneal dystrophy [11], monoclonal gammopathy [12], corneal biomechanics [13,14], and KC [2,15,16,17]. In addition, densitometry is frequently used to assess corneal transparency at different stages of KC [15,18]; therefore, it is useful to understand the effects of any artefacts inherent in the measurement system.

The current study explores and analyses artefacts in densitometry maps generated by the Pentacam rotating Scheimpflug camera system (Oculus, Inc., Wetzlar, Germany), where the densitometry measurements are expressed in a grey scale from 0 to 100. Lower values represent greater transparency, and a score of 100 means a totally opaque cornea. For the analysis, a set of custom-built MATLAB codes (MathWorks Inc, Natick, MA, US) were written to automatically import Pentacam data for N and KC corneas in a double-precision numerical format up to 64-bit, then they were processed without manual human intervention, from raw data to final figures, to eliminate human factors during digital data processing entirely.

## 2. Methods

### 2.1. Subject Data Collection and Processing

This retrospective study utilised fully anonymised records from Brazil’s Hospital de Olhos Santa Luzia, including N and KC patients. The analyses were conducted following the standards of the Declaration of Helsinki and consented to by the ethical committee board of the Federal University of São Paulo (UNIFESP/SP 2020 # 4.050.934). Subjects were excluded where there was a history of ocular diseases (other than KC), history of trauma or ocular surgery, and intraocular pressure (IOP) above 21 mmHg as measured by Ocular Response Analyser (Reichert Technologies, Depew, NY, USA). If the subjects wore contact lenses, they were required to stop wearing them for a period of time before topography measurement [19]; soft contact lens wearers were asked to remove lenses two weeks prior to measurements and rigid, gas-permeable (RGP), four weeks prior.

The data collected in the study were collected by scanning one randomly selected eye of each participant. Where a subject had only one confirmed KC eye, this eye was chosen. At least three successive scans were taken for each subject, with approximately a period of roughly half a minute between them.

The measurements were repeated until three scans with at least 90% instrument-generated quality factors were achieved. Only scans with an examination quality status identified as “OK” and error rank “0” were considered and analysed. Room lights were switched off during data acquisition, and the Pentacam computer screen was directed away from the participant’s face to reduce interference from external light scattering. Participants were asked to place their chin on the chinrest and their forehead on the foreheadrest and then to fixate on a target at the centre of the instrument camera while the operator carried out instrument adjustments using the joystick. The subjects were asked to blink and reposition their head between each shot while the instrument was pulled back fully and then realigned.

In total, 458 N eyes aged 35.6 ± 15.8 (10–87) and 314 KC eyes aged 31.6 ± 10.8 (10–72) were included in the current study. Table 1 refers to the clinical characteristics of the study participants. Additionally, the severity of the KC cases was classified from the Pentacam maps to form subgroups according to TKC index grading. In the current study, Stage 1 refers to a TKC of 1 or less (21.5%), Stage 2 refers to a TKC of either 1–2 or 2 (37.9%), Stage 3 refers to a TKC of either 2–3 or 3 (28.2%), and Stage 4 refers to a TKC of either 3–4 or 4 (12.4%). Table 1 shows the ranges of the multivariate index of Bad D, where the inclusion criterion for the KC participants was a clear presence of KC with no previous ocular procedures, such as collagen crosslinking and an index of Bad D more than 1.5 D/mm [20].

### 2.2. Measurement of Corneal Densitometry

**Figure 1:**
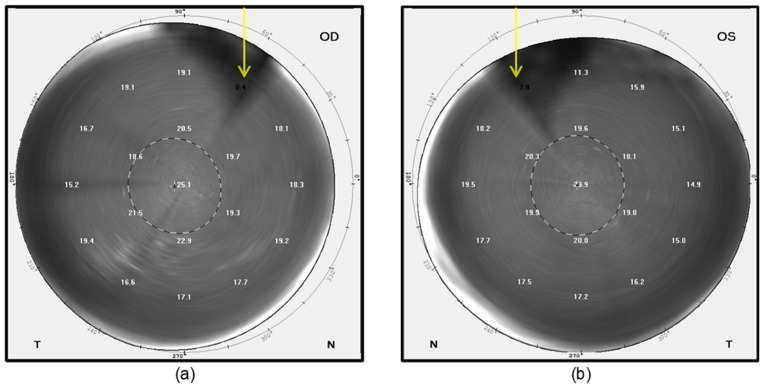
Sample densitometry maps in NGSU for 61-year-old male participant fellow eyes as displayed by the Pentacam software—the yellow arrows point to artefact-affected areas as visually identified. (**a**) Oculus Dexter (OD) or right eye; (**b**) Oculus Sinister (OS) or left eye. In this figure, T stands for temporal, and N stands for nasal.

The Pentacam system’s software (1.27r13) measures the corneal depth over three layers; the anterior layer (120 µm), the posterior layer (60 µm), and the central layer, which comprises the rest of the corneal thickness. The standard Pentacam settings allow the device to capture 25 cross-section images over evenly spread meridians. These images are interpolated through an image signal processor integrated within the Pentacam software to create a densitometry map over a diameter up to 12 mm in the post-measurement processing stage. The Pentacam software-driven map is calibrated and expressed in normalised gray-scale units (NGSU). The intensity values cover 256 brightness levels (0 for black to 255 for white) and are normalised by the number of brightness levels to be within the 0 to 100 range; therefore, a minimum light scatter of 0 (black) represents no light scattering, indicating maximum transparency, and a maximum light scatter of 100 (white) indicates minimum transparency [3], Figure 1.

### 2.3. Processing Corneal Densitometry Measurements

**Figure 2:**
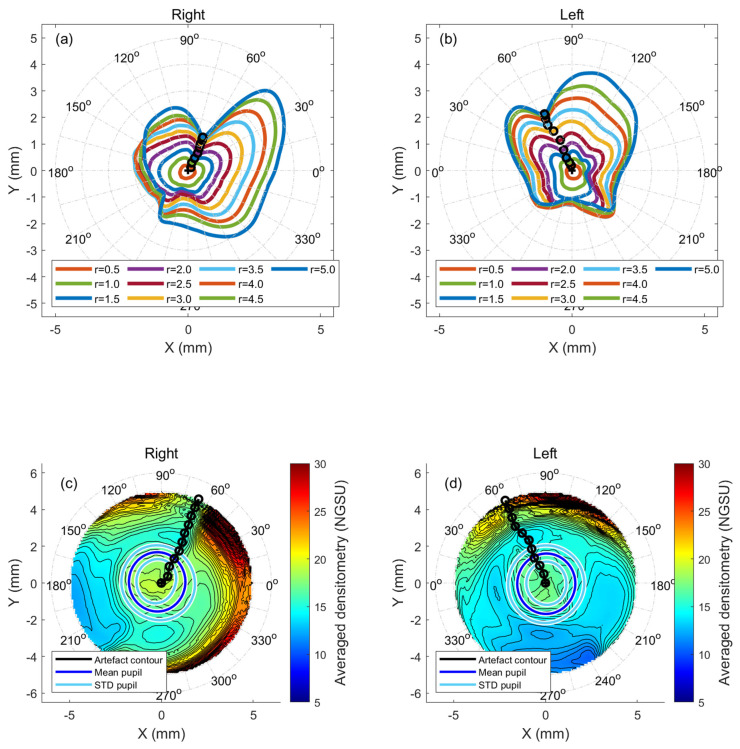
Normal eyes normalised radial summation of densitometry, (**a**) right eyes at different radii in mm, (**b**) left eyes at different radii in mm, (**c**) averaged cornea densitometry for right eyes with artefact contour line marked in black, and (**d**) same as c but for left eyes.

**Figure 3:**
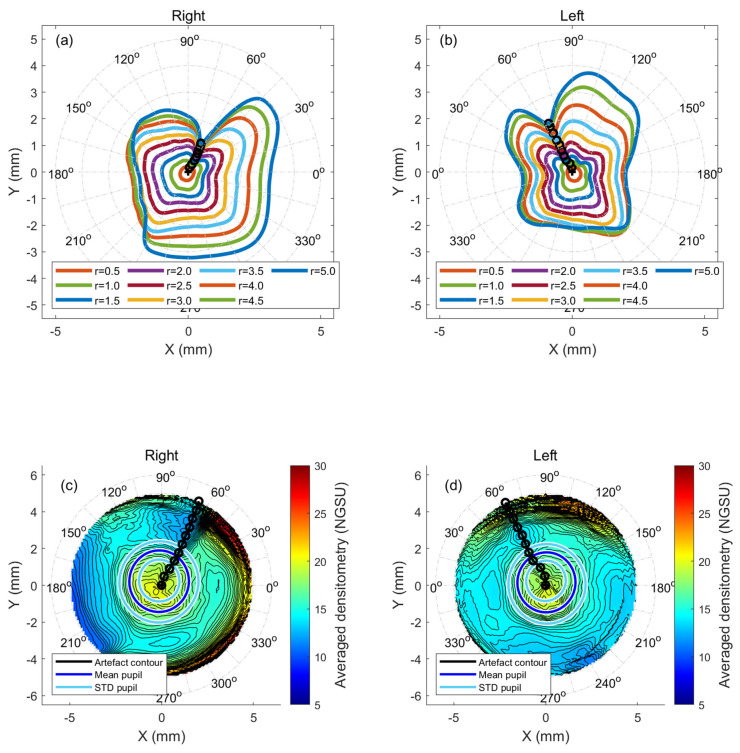
KC eyes normalised radial summation of densitometry (**a**) right eyes at different radii in mm, (**b**) left eyes at different radii in mm, (**c**) averaged cornea densitometry for right eyes with artefact contour line marked in black, (**d**) same as c but for left eyes.

Densitometry measurements were constructed collectively in a three-dimensional matrix for each cornea before being averaged as a map. Pentacam densitometry data were extracted over the corneal apex, as a central point, with a mesh grid covering −7.0 to 7.0 mm in 0.1 steps in both nasal-temporal and superior-inferior directions with missing elevation values around the corners and edges, set to NaN (Not a Number). A fully 100%-computerised custom-built MATLAB code was written primarily for this study to allow comprehensive, repeatable, automated analyses. Before processing them separately, the code read the comma-separated values (CSV) files of all participants’ right or left corneas. Although no fellow eyes of the same participant were included, right and left eyes were initially analysed separately to ensure that variances between the right and left eyes could be detected. To compare KC and N corneas, left eye data were flipped around the superior–inferior axis to allow a proper comparison between the nasal and temporal data. This combination facilitated the drawing of overall conclusions regarding any artefacts’ effect on using densitometry as a tool for KC detection. Different maximum radii values of 0.5 mm to 5.0 mm, centred at the corneal apex, in 0.5 mm steps, were considered in the analyses to reflect the investigated artefact effect over the corneal radius. The radial summation of each densitometry measurement map was calculated over 360° meridians and then normalised to the range [0, 1] before being multiplied by the radius where the densitometry was calculated. The resulting marker is named the “normalised radial summation of densitometry” (NRSD) and was used for further processing. Minimum NRSD values were automatically identified, located, and their angular positions were recorded. Identified NRSD angular positions were linked to calculation radii and then connected on the densitometry map by a black line to indicate where the ultimate distortion occurs (see Figure 2 and Figure 3).

### 2.4. KC Cone Centre Location and Boundary Analyses

The current study characterises the KC corneas’ cone centre and size by an updated version of a previously published method [21] where a best-fit ellipsoid was used as a reference surface instead of a perfect sphere reference to counteract the effect of corneal astigmatism while identifying the KC cone effect. The method can be outlined in three stages as follows. First, the corneal surface was levelled using a first-order Zernike polynomial fitted plane following the method outlined in previous work [22], and then the resultant surface was best-fitted to an ellipsoid using a minimum least squared error method. Secondly, the distances from the centre of the eye’s best-fit ellipsoid to each point on the corneal surface were calculated by switching to spherical coordinates with the origin at the best-fit ellipsoid’s centre; hence, the height data were presented by their X and Y cartesian coordinates and their spherical coordinate radii as a third dimension. Finally, the radii of the best-fit ellipsoid were subtracted from the spherical coordinate radii that represent the corneal height data to obtain a flattened elevation map. This method of describing the corneal surface separates the natural curvature of the cornea from the abnormal curvature resulting from the KC cone.

The anterior and posterior centres of the KC cone were then detected as the highest points in the spherical height maps of the corneal surfaces. The cone boundary was detected by investigating an area with a 2 mm radius around the cone centre by dividing it into 360 meridians with 1° spacing to attain maximal coverage. Then, the second derivative of each of these meridians was used to detect the rate of change in the surface tangent. A rapid change in this rate identified the position where the corneal curvature transforms from the steep area of pathology within the KC cone to a more regular corneal shape area beyond it. Joining these identified positions of KC cone edges in the 360 meridians formed the KC cone boundary for each KC eye.

In the current study, the averaged KC cone boundaries were approximated to circles and calculated by fitting the areas of pathology to circular shapes, hence calculating the radii and then averaging them. A histogram plot for KC cone centres was then introduced with a bin size of 0.5 mm to represent the distribution of KC cone centres. Averaged densitometry analyses by KC severity were conducted twice; once within the KC averaged cone boundary in the KC group and once covering the whole measured portions of the KC group’s corneas.

## 3. Statistical Analyses

The statistical analyses of the current study were performed via MATLAB Statistics and Machine Learning Toolbox software (R2023b). The built-in MATLAB function “ttest2” was utilised for two-sample *t*-tests to return *p*-values and test decisions for the null hypothesis in a binary format. A confidence level of 95.0% was used to set the null hypothesis to examine the interpretations of the results based on statistical indications. Using the Kolmogorov–Smirnov test [23], the normal distribution of the samples was confirmed before each of the two-sample *t*-tests were implemented to explore whether the data set couples were significantly different and to check whether the evaluated results characterised an independent data record. Within the closed period of 0.0 to 1.0, the probability *p*-value higher than 0.05 indicates that the null hypothesis cannot be rejected [24]. Significance in the current study was reported for every point on the corneal surface; therefore, full significance maps, not singular points, were presented to compare regional variations in the corneal surface densitometry measurements.

## 4. Results

Plotting the NRSD at the radii of 0.5 mm to 5.0 mm showed the radial distribution of densitometry around the cornea in both N eyes (Figure 2a,b) and KC eyes (Figure 3a,b). When the minimum radial summations of the densitometry contour lines were joined, the results of both the N and KC groups showed an apparent disturbance in the radial densitometry in nasal-superior directions in the right and left eyes. Angles were always measured from the nasal axis to properly compare the right and left eyes. Right eyes in the N group recorded an artefact contour at 66° at a radius of 5.0 mm, while the KC group recorded 64° at the same radius. For N’s left eyes, the artefact contour pointed to 66° while the value was 65° in KC’s left eyes. A range of radii, varying from 0.5 mm to 5 mm, showed that the artefact contour line angles slightly varied within the central cornea before becoming steady towards the periphery, where they always pointed to the nasal-superior direction, Table 2.

The averaged densitometry among the N and the KC groups showed a trend supporting the findings obtained from the artefact contour lines. The distortion in the nasal-superior direction was evident in both groups and with right and left eyes (see Figure 2c,d and Figure 3c,d).

When comparing males and females, KC males within the study recorded slightly lower average densitometry (12.80 ± 3.04%) than females (13.51 ± 2.93%), which was not significant (*p* = 0.1192). In terms of Bad D, females demonstrated an average of 8.22 ± 5.57 and males a slightly lower average of 7.95 ± 4.36, with no significant difference between the two groups (*p* = 0.7216). There was also no significant difference in the average densitometry between KC males and females (*p* = 0.1192). TKC was 2.13 ± 0.98 among males and 1.82 ± 1.07 among females, with a significant difference (*p* = 0.0448). Similarly, there was no significant difference (*p* = 0.8951) in the average densitometry among the N group between males (10.10 ± 3.26%) and females (10.03 ± 4.20%).

Age analyses revealed that the average densitometry was weakly correlated with age among the N group (R = 0.04), but moderately correlated with age among the KC group (R = 0.63).

**Figure 4:**
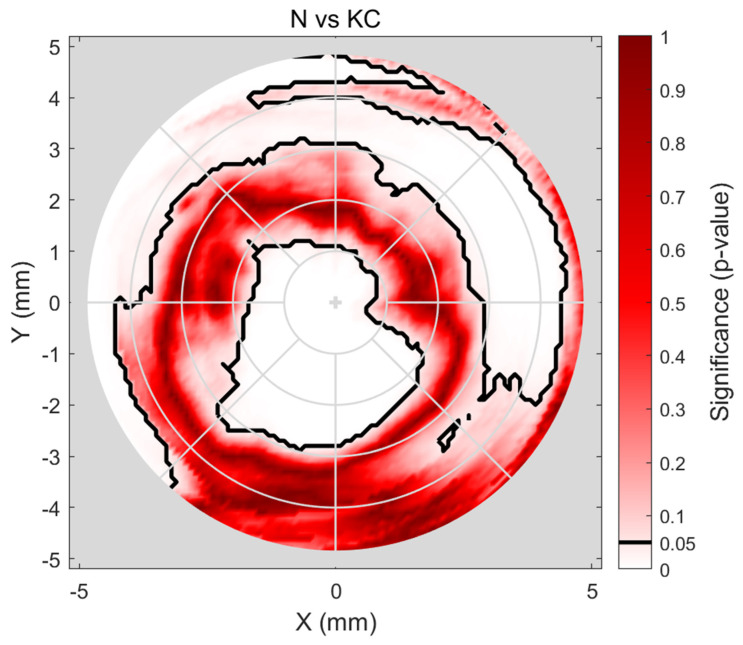
Investigating the significance of the difference between N and KC eyes’ densitometry. The black contour marks *p* = 0.05 significance boundary, where values within this limit indicate statistical insignificance.

Significance analysis to evaluate the difference in densitometry among N and KC groups showed significance in the central (~1 mm diameter) area (*p* < 0.001), insignificance within the corneal ring diameters ~3 to ~6 mm (*p* ≈ 0.75), and then, in the periphery, the significance covers all directions apart from the inferior and nasal-inferior sections (*p* < 0.001). The results are displayed on a map representing the whole cornea where black boundaries are used to display *p* = 0.05, Figure 4.

**Figure 5:**
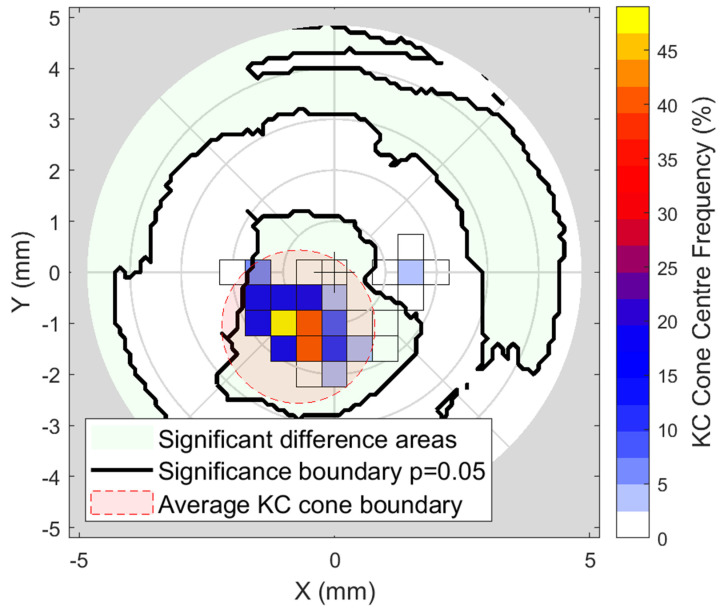
Frequency plot of the KC cone centres with the N vs. KC significance areas shaded in light green and averaged KC cone boundary coloured in light red.

**Figure 6:**
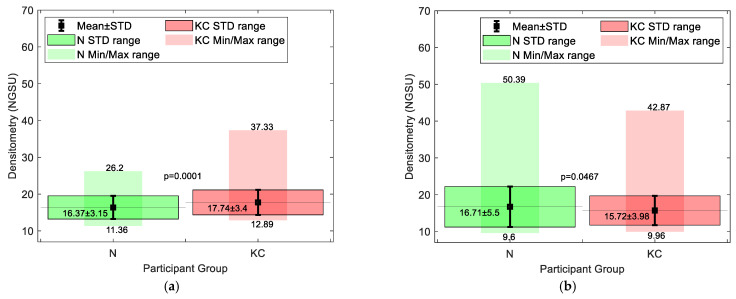
Averaged densitometry (**a**) within the KC averaged cone boundary in the KC group compared to the equivalent area among the N group and (**b**) covering the whole KC group corneas compared to the N group.

Cone centre frequency plots indicated that most cones (≈50) in the KC group were approximately centred at X = −1.25 mm, Y = −1.25 mm towards the nasal-temporal side, Figure 5. It can be seen that most cones are within the central area, where there is a significant difference in densitometry between the N and KC groups. When the averaged densitometry was compared between the N group and the KC group, within the averaged KC cone area of pathology, the KC group recorded 17.74 ± 3.4 NGSU, slightly more than the N group, which recorded 16.37 ± 3.15 NGSU. However, when the averaged densitometry was compared over the entire cornea, the N group recorded 16.71 ± 5.5 NGSU, slightly higher than the KC group, which recorded 15.72 ± 3.98 NGSU, Figure 6. Both comparison methods showed significant differences between the N and the KC groups, but overlap between the standard deviation of the two groups was evident.

**Figure 7:**
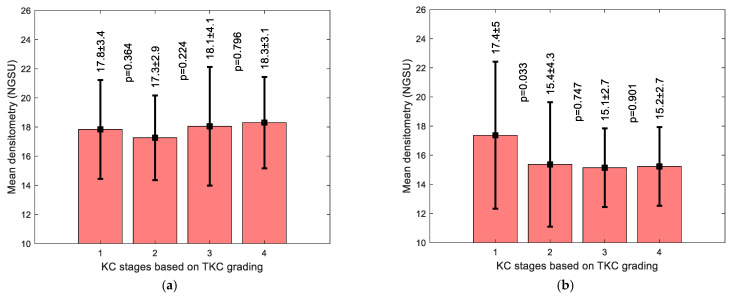
Averaged densitometry subgroup analysis by KC severity classified by TKC index showing averaged densitometry (**a**) within the KC averaged cone boundary in the KC group and (**b**) covering the whole KC group corneas. Following the TKC grading method, stage 1 (TKC = 0–1,1), stage 2 (TKC = 1–2,2), stage 3 (TKC = 2–3,3), and stage 4 (TKC = 3–4,4).

**Figure 8:**
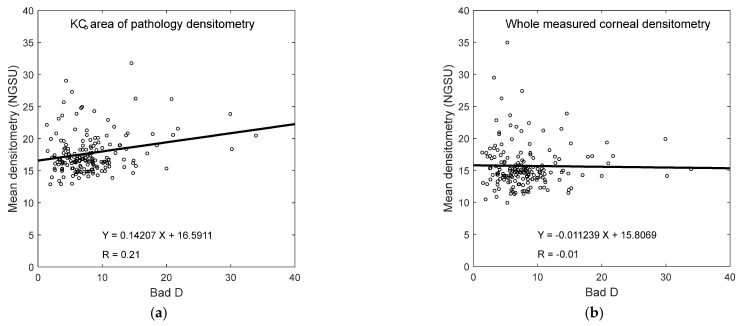
Correlation between Bad D index and averaged densitometry (**a**) within the KC averaged cone boundary in the KC group and (**b**) covering the whole KC group corneas.

Averaged densitometry KC subgroup analysis showed an increase in the NGSU with the rise in KC severity, except for stage 1 when the KC area of pathology was considered, Figure 7a; however, when the whole cornea was considered, Figure 7b, there was no clear densitometry trend. When the correlation between the Bad D index and the average densitometry was investigated, a weak correlation was found between the Bad D index and NGSU either within the KC area of pathology, Figure 8a, or when the whole measured cornea was considered, Figure 8b; however, a slightly better correlation was recorded within the KC area of pathology.

## 5. Discussion

**Figure 9:**
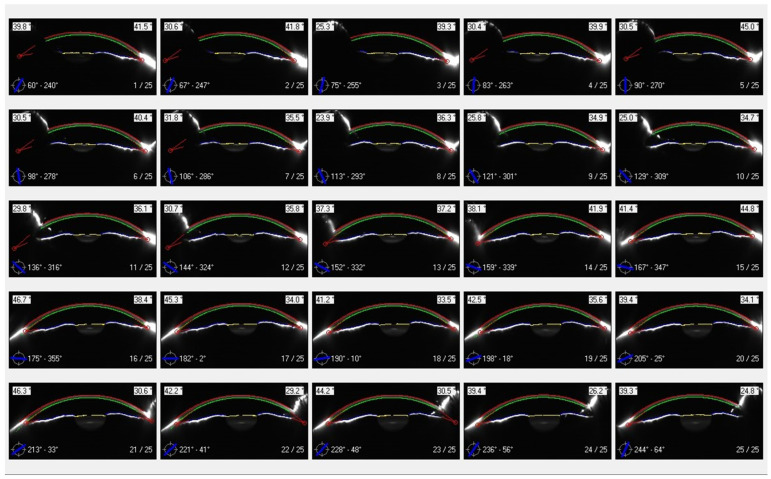
A typical set of images from the Scheimpflug Pentacam demonstrating superior shadowing effects from the upper eyelid and brow and the inequality of backscatter from the iris. The participant is 45 years old from the N group. The green contour indicates the detected corneal anterior surface and the blue line shows the image’s cross-sectional angular position.

The use of corneal densitometry to assess corneal disorders [25,26] and diseases [2,27] has been reported in several studies. In addition to KC, it has been suggested as a tool for evaluating refractive surgery [28,29], trabeculectomy [30], and corneal collagen crosslinking [31]. As unquantified light scattering due to reflections from the limbal area, intraocular lens, corneal tilt, and potentially facial anatomy could impact corneal densitometry readings, the current study investigated a noticed phenomenon in Pentacam densitometry maps where an artefact appeared to present in a nasal-superior direction in each eye, Figure 1. Figure 9 shows a typical set of Scheimpflug images taken over a range of 25 meridians. It can be seen that the area below the top lid is in shadow for many of the images, so it was difficult for the Pentacam software to attain optimal edge detection. In addition, the images show significantly reduced backscatter from the superior iris, whilst the inferior iris consistently demonstrates a reasonable amount of scatter. The combination of these two effects may result in an apparently increased transparency towards the periphery, resulting in the observed artefact.

In investigating these artefacts, other potential influences on densitometry were investigated. It has been previously found that densitometry does not appear to correlate with either keratometry or refractive power [22]. Although an increase in corneal densitometry has been found in healthy eyes with age [32], the age analysis of the N and KC groups showed that the average densitometry was weakly correlated with age among the N group, but moderately correlated with age among the KC group. This may be linked to the severity of KC being more likely to be higher with age, as it is a progressive condition [13]. There was no significant difference in the average densitometry between males and females among both N and KC groups. It is possible that the ethnicity of the participants may affect densitometry, but in this retrospective study, this was not easy to define as the Brazilian population is mixed with European, African, and Asian countries contributing to the genetic pool in addition to Native American, with variations depending on the region [33].

In terms of the instrument itself, like many parameters measured by a digital device, densitometry is affected by systematic errors and digital noise; therefore, a careful assessment of the maps produced by such devices and how they relate to the relative orientation of the slit-light source of the investigated phenomenon is needed when drawing conclusions about the conditions being studied. The quantitative analyses showed that the maximum densitometry artefact always points to the nasal-superior direction, regardless of the eye (right or left) or the eye condition (N or KC). Considering the fact that the vast majority of KC cones were found to be on the inferior-temporal side [21], opposite to the direction of the maximum densitometry artefact, which is nasal-superior according to the current study, this artefact should not affect the use of densitometry for assessing KC within the KC area of pathology. The orientation of densitometry artefacts in the analyses indicates that the systematic artefact effect in the Pentacam densitometry maps could result from the variable distribution of shadows and light reflections and should be considered when assessing densitometry maps. One of the possible limitations is that although the time of day of measurements is important when considering densitometry, as there is a diurnal variation in corneal thickness [34], this was not possible due to the retrospective nature of the study.

When comparing the N and KC groups from 2D densitometry maps, it was clear that a significant difference (*p* = 0.0001) in densitometry occurs around the KC area of pathology compared to the same area in the N group. This result indicates that the existence of the KC cone increases the light scattering within the area of pathology. These are anticipated results as it is known that the progress of KC involves a substantial localised interlamellar and intralamellar dislocation and slippage that cause cornea thinning associated with steepened corneal curvature [35], hence the cause of the rise in light scattering.

Conversely, when the whole corneal densitometry was compared between the N and the KC groups, N corneas recorded higher overall scattering than KC corneas (*p* = 0.0467). This indicates that analysing the densitometry of the whole corneal surface of KC eyes, that is, averaging the healthy with the pathologically affected cornea, including the superior-nasal densitometry artefacts, reduces the KC detectable effect if the averaged NGSU densitometry is to be used as a KC identifier or classifier.

Although N versus KC comparisons showed significant differences between the N and KC groups’ densitometries, the overlap of the standard deviation (STD) range (see Figure 6) suggests that it is clinically difficult to use densitometry measurements alone to distinguish between N and KC corneas, as the difference between the two is not large enough for a clinically clear indication. Within the KC area of pathology, averaged densitometry subgroup analysis showed a slightly increasing trend in NGSU densitometry scattering in successively more severe KC stages; however, no significance was noticed among KC stages *p* > 0.05. This slight trend disappeared when the whole measured cornea was investigated against different KC stages, which indicates that locating the KC cone is vital to recognise small trends in densitometry.

In conclusion, the results of this investigation show that the densitometry artefact of the Scheimpflug-based system of Pentacam is systematic and disturbs both N and KC corneas, but lies outside the KC cone area of pathology; therefore, it should not affect the capability of densitometry to be used in the evaluation of KC corneas in supplementary applications, theoretically. Despite this weak ability to be used as a sole classifier, densitometry could be used to enhance artificial intelligence (AI) models of identifying or classifying KC when combined with other topographic and biomechanical metrics. Based on the current study, it is recommended that densitometry in the inferior-temporal area of the cornea should be more highly weighted than the superior-nasal as the former is the most common area of pathology and the latter is more likely to be affected by artefacts.

Finally, future work will consider the possible effect of the top lid shadow artefacts on other maps, such as axial, tangential, and refractive power maps.

## Figures and Tables

**Table 1:** Clinical features of the participants as measured by the Pentacam system.

Clinical Classification	Normal (N)		Keratoconic (KC)	
	Mean±STD	Min:Max	Mean±STD	Min:Max
Minimum corneal thickness (µm)	550 ± 33	492:660	466 ± 39	307:568
Flat radius of curvature in the central 3 mm zone K1 (D)	42.6 ± 1.4	39.4:46.6	44.7 ± 3.1	36.7:55.3
Steep radius of curvature in the central 3 mm zone K2 (D)	43.8 ± 1.5	40.3:47.9	48.4 ± 3.7	41.8:59.4
Topographical KC classification TKC (level)	NA	NA	2.1 ± 0.7	1:3.5
Index of Bad D (D/mm)—less than 1.5 is normal [20]	0.4 ± 0.5	−0.9:1.4	7.0 ± 3.3	1.5:20.8

**Table 2:** Artefact contour line angles as detected in different radii with 0° pointing to the nasal side and 90° pointing to the superior side.

	Right Eye Angles (°)		Left Eye Angles (°)	
r (mm)	N	KC	N	KC
0.5	69	74	45	84
1.0	59	60	69	68
1.5	63	63	66	62
2.0	61	62	67	64
2.5	62	62	67	65
3.0	65	64	63	64
3.5	65	64	62	64
4.0	66	65	63	64
4.5	66	65	64	64
5.0	66	66	64	65

## Data Availability

The data presented in this study are available on request from the corresponding author.

## Abbreviations

CSV	Comma-separated values file
IOP	Intraocular pressure
KC	Keratoconic
N	Normal
NaN	Not a number
NGSU	Normalised grey-scale unit
NRSD	Normalised radial summation of densitometry
RGP	Rigid gas-permeable
STD	Standard deviation
